# The Association Between Adherence to Mediterranean, DASH, and MIND Diets and the Risk of Breast Cancer: A Case–Control Study in Iranian Women

**DOI:** 10.1155/ijbc/7418288

**Published:** 2026-04-17

**Authors:** Farnush Bakhshimoghaddam, Nikoo Mohtadi, Abolfazl Haghighatkhah, Ahmad Zare Javid, Maedeh Raeisi Zadeh, Samira Razzaghi, Mina Moradpour, Hamidreza Razmi

**Affiliations:** ^1^ Student Research Committee, Ahvaz Jundishapur University of Medical Sciences, Ahvaz, Iran, ajums.ac.ir; ^2^ Nutrition and Metabolic Diseases Research Center, Clinical Sciences Research Institute, Ahvaz Jundishapur University of Medical Sciences, Ahvaz, Iran, ajums.ac.ir; ^3^ Department of Nutrition, School of Allied Medical Sciences, Ahvaz Jundishapur University of Medical Sciences, Ahvaz, Iran, ajums.ac.ir; ^4^ Department of Biostatistics and Epidemiology, School of Public Health, Ahvaz Jundishapur University of Medical Sciences, Ahvaz, Iran, ajums.ac.ir; ^5^ Department of Clinical Oncology, Golestan Hospital, Ahvaz Jundishapur University of Medical Sciences, Ahvaz, Iran, ajums.ac.ir

**Keywords:** breast cancer, diet, Dietary Approaches to Stop Hypertension, Mediterranean, menopause

## Abstract

**Purpose:**

Breast cancer is a leading cause of cancer‐related morbidity and mortality among women worldwide. This study was aimed at investigating the association between adherence to the Mediterranean diet (MD), Dietary Approaches to Stop Hypertension (DASH), and Mediterranean‐DASH Intervention for Neurodegenerative Delay (MIND) diets and the odds of developing breast cancer.

**Methods:**

A hospital‐based case–control study was conducted with 106 histopathologically confirmed breast cancer cases and 107 age‐matched controls. Dietary intake was assessed using a validated 147‐item food frequency questionnaire. Adherence scores for the MD, DASH, and MIND diets were calculated and categorized into tertiles. Multivariable logistic regression models were used to estimate odds ratios (ORs), adjusting for age, body mass index, waist‐to‐hip ratio, physical activity, education, employment, and family history of cancer.

**Results:**

After full adjustment, compared to the lowest tertile, women in the highest tertile of MD, DASH, and MIND diet adherence had a 75% (OR = 0.25, 95% CI: 0.10–0.64), 76% (OR = 0.24, 95% CI: 0.09–0.62), and 72% (OR = 0.28, 95% CI: 0.11–0.75) reduced odds of breast cancer, respectively. All trends were statistically significant. However, stratification by menopausal status revealed no significant associations within pre‐ or postmenopausal subgroups.

**Conclusion:**

Greater adherence to the MD, DASH, and MIND diets is associated with a substantially lower likelihood of breast cancer in Iranian women. These findings highlight the potential of healthy dietary patterns as a modifiable factor for breast cancer prevention in this population.

## 1. Introduction

Breast cancer remains the most frequently diagnosed malignancy and a leading cause of cancer‐related mortality among women worldwide, with an estimated 2.3 million new cases and 685,000 deaths reported in 2020 [1]. The global incidence of breast cancer continues to rise, particularly in regions undergoing rapid socioeconomic transition, underscoring the urgent need for effective preventive strategies [2]. While nonmodifiable risk factors such as age, genetic predisposition, and reproductive history contribute significantly to disease etiology, growing evidence highlights the pivotal role of modifiable lifestyle factors, particularly diet, in breast cancer pathogenesis [3].

In nutritional epidemiology, the analysis of dietary patterns has gained prominence over the examination of isolated nutrients or foods, as it more accurately reflects the complexity of dietary habits and their synergistic effects on health outcomes [4]. Among various dietary patterns, the Mediterranean diet (MD), characterized by high consumption of fruits, vegetables, legumes, whole grains, nuts, and olive oil, along with moderate fish intake and limited red and processed meats, has been extensively studied for its potential protective effects against chronic diseases, including breast cancer [5]. A recent umbrella review of systematic reviews and meta‐analyses suggested that adherence to the MD is associated with a reduced risk of breast cancer, particularly among postmenopausal women [6]. Similarly, the Dietary Approaches to Stop Hypertension (DASH) diet, which emphasizes similar food groups while specifically limiting sodium, sugar‐sweetened beverages, and sweets, has also demonstrated inverse associations with breast cancer risk in several observational studies [7].

More recently, the Mediterranean‐DASH Intervention for Neurodegenerative Delay (MIND) diet has emerged as a hybrid dietary pattern that combines elements of both the MD and DASH diets, with a specific emphasis on neuroprotective foods such as green leafy vegetables, berries, nuts, and whole grains [8]. While the MIND diet has been robustly associated with reduced risk of cognitive decline and neurodegenerative diseases [8, 9], its relationship with cancer outcomes, including breast cancer, remains underexplored. To date, only a limited number of studies have investigated the association between MIND diet adherence and breast cancer risk, with inconsistent findings reported [10, 11]. This highlights a significant gap in the literature regarding the potential role of this promising dietary pattern in breast cancer prevention.

Furthermore, most existing evidence derives from Western populations, and data on the association between these dietary patterns and breast cancer risk in Middle Eastern populations, particularly Iranian women, are scarce. Given the distinct dietary habits, genetic background, and breast cancer epidemiology in this region, population‐specific investigations are crucial to inform targeted preventive strategies.

Therefore, this hospital‐based case–control study was aimed at evaluating the association between adherence to three dietary patterns, including the MD, DASH, and MIND diets, and the risk of breast cancer among Iranian women. We hypothesized that greater adherence to these healthy dietary patterns would be associated with a lower likelihood of breast cancer, thereby providing valuable insights for dietary recommendations and public health initiatives aimed at reducing the breast cancer burden in this population.

## 2. Materials and Methods

A hospital‐based case–control study was conducted among Iranian women aged 18 years and above who were referred to Golestan Hospital, a tertiary care center in Ahvaz, between December 2023 and June 2024. The case group included women with a histopathologically confirmed diagnosis of breast cancer, identified through the hospital′s Radiotherapy and Oncology Department. The control group included patients from other hospital departments with nononcological conditions unrelated to long‐term dietary patterns, such as diabetes, gastrointestinal disorders, and chronic renal disease. Conditions among controls included acute surgical presentations, orthopedic and trauma disorders, and otolaryngological diseases requiring hospital stays of up to 3 days. All control participants had maintained a stable body weight (± 3 kg) over the preceding 6 months and reported no significant dietary changes within the past year. Cases and controls were frequency‐matched by age (± 5 years). Data collection questionnaires were administered at a mean of 1.6 years (± 2.7) following breast cancer diagnosis (range: 0.5–5 years). Of the 148 eligible control subjects invited, 125 participated, resulting in an 84.5% response rate. Following the exclusion of 18 controls and 14 cases due to missing data or energy intake values outside the plausible range (mean ± 3 SD), the final analysis comprised 106 cases and 107 controls.

## 3. Demographic and Clinical Data Collection

Demographic and clinical data were obtained through face‐to‐face interviews conducted by trained personnel using structured questionnaires. The collected information included lifestyle characteristics (e.g., physical activity and smoking), socioeconomic status, hormonal and reproductive history, and family history of breast and other cancers.

### 3.1. Anthropometric Measurements

All anthropometric measurements were taken with participants in light clothing and without shoes. Body weight and height were measured using a digital scale (to the nearest 100 g) and a stadiometer (to the nearest 0.1 cm), respectively. Body mass index (BMI) was calculated as weight in kilograms divided by height in meters squared (kg/m^2^). Waist and hip circumferences were measured to the nearest 0.1 cm using a nonstretch tape measure. Waist circumference was taken at the level of the umbilicus, and hip circumference was measured at the point of maximum gluteal protrusion. The waist‐to‐hip ratio (WHR) was derived from these measurements.

### 3.2. Physical Activity Assessment

Physical activity levels were assessed using the short form of the International Physical Activity Questionnaire (IPAQ). Outcomes are expressed as metabolic equivalent minutes per week (MET‐min/week). This instrument has been validated for use in an Iranian adult population [12].

### 3.3. Dietary Assessment

Dietary intake was assessed using a 147‐item semiquantitative food frequency questionnaire (FFQ) specifically designed and validated for the Iranian population [13, 14]. This instrument has demonstrated reliability and validity in capturing habitual dietary patterns among Iranians. Participants reported their consumption frequency (daily, weekly, or monthly) and portion sizes for each food item over the preceding year. Responses were converted to estimate daily intake quantities. Portion sizes, calculated using standard household measures, were converted to gram weights [15]. Subsequently, daily intakes of energy, protein, carbohydrates, fat, and fiber were computed for each participant using the Nutritionist IV software.

## 4. MD Score Calculation

Adherence to the MD was assessed based on the methodology of Trichopoulou et al. [5]. The score comprises nine components: fruits and nuts, vegetables, legumes, whole grains, fish, the ratio of monounsaturated to saturated fatty acids (MUFA: SFA), moderate alcohol consumption (wine), meat (including red meat, poultry, and processed meats), and dairy products. In the current study, the alcohol component was omitted from the MD score. This modification was necessary because alcohol consumption was not assessed in our dietary questionnaire, reflecting its rarity in the study population due to cultural and religious norms. The scoring protocol was as follows: for components beneficial within the MD (fruits and nuts, vegetables, legumes, whole grains, fish, and MUFA: SFA ratio), participants received 2 points for consumption in the highest tertile, 1 point for the middle tertile, and 0 points for the lowest tertile. Conversely, for components atypical of the diet (meat and dairy), the scoring was reversed: 0 points for the highest tertile, 1 point for the middle tertile, and 2 points for the lowest tertile. Therefore, the overall MD score for each participant was calculated as the sum of points across all eight components, yielding a theoretical range of 0 (minimal adherence) to 16 (maximal adherence).

## 5. DASH Diet Score Calculation

Adherence to the DASH diet was assessed by constructing a score based on eight components: high intake of fruits, vegetables, nuts, legumes, and whole grains, and total dairy products and low intake of red and processed meats, sugar‐sweetened beverages, sweets, and sodium [7]. For components where high intake is encouraged (fruits, vegetables, nuts, legumes, whole grains, and dairy), a score of 5 was assigned to the highest consumption quantile, decreasing to 1 for the lowest. Conversely, for components where low intake is encouraged (red and processed meats, sugar‐sweetened beverages and sweets, and sodium), the scoring was inverted: the highest quantile received a score of 1, and the lowest decile received a score of 5. The overall DASH score for each participant was calculated by summing the scores across all eight components, yielding a total score ranging from 5 (lowest adherence) to 50 (highest adherence).

## 6. MIND Diet Score Calculation

The MIND diet score was adapted from the original 15‐component scoring system [8], which comprises 10 “brain‐healthy” food groups (green leafy vegetables, other vegetables, nuts, berries, beans, whole grains, fish, poultry, olive oil, and wine) and five “brain‐unhealthy” food groups (red meats, butter and stick margarine, cheese, pastries and sweets, and fried/fast food). The wine component was excluded from the MIND score calculation due to insufficient data in our dataset, as its consumption is uncommon in the study population. Therefore, a modified score based on the remaining 14 components was used. For these 14 components, participants were categorized into tertiles based on their intake levels. For the eight brain‐healthy components, participants received a score of 1 for the highest tertile, 0.5 for the middle tertile, and 0 for the lowest tertile. Conversely, for the five brain‐unhealthy components, the scores were inverted: 1 was assigned to the lowest tertile, 0.5 to the middle tertile, and 0 to the highest tertile. The overall MIND diet score for each participant was calculated by summing the scores across all 14 components, yielding a total score ranging from 0 to 14, with higher scores indicating greater adherence.

## 7. Statistical Analysis

The mean (standard deviation) and the frequency (percentage) were used for continuous variables and categorical variables, respectively. The Kolmogorov–Smirnov test was used to assess the normality of the constant data. Cases and controls were compared on general characteristics using independent‐samples *t*‐tests and chi‐squared tests. To account for total energy intake and examine diet composition independently, we used the residual method to energy‐adjust the intakes of all food groups and nutrients relevant to the MD, DASH, and MIND score components [16]. Participants were then categorized according to tertiles of the MD, DASH, and MIND scores. One‐way ANOVA was used to examine differences in continuous variables across the three tertiles of the dietary scores. Additionally, the chi‐squared test was used to evaluate the distribution of categorical variables across tertiles of MD, DASH, and MIND dietary scores. We used binary logistic regression in adjusted models to assess the association between dietary adherence scores and breast cancer. In the adjusted model, we adjusted for participants′ age, BMI, WHR, physical activity (low, moderate, and high), education (illiterate, low, moderate, and high), employment status (housekeeper and employee), and family history of breast (yes and no) or other cancer (yes and no). Participants in the first tertile of dietary scores were used as the reference group in all analyses. Furthermore, menopausal status (premenopausal vs. postmenopausal) was stratified in the logistic regression analysis. All statistical analyses were performed using SPSS software. In this study, the significance level was *p* < 0.05.

## 8. Result

The baseline characteristics of the study participants, stratified by case and control status, are presented in Table 1. Cases compared to control groups had a significantly higher WHR (0.89 ± 0.07 vs. 0.83 ± 0.11, *p* < 0.001) and were more likely to be postmenopausal (41.5% vs. 13.1%, *p* < 0.001). Furthermore, cases had a lower education level (*p* = 0.04), were more likely to be housekeepers (87.7% vs. 70.1%, *p* = 0.002), and had a higher prevalence of family history of breast cancer (26.0% vs. 10.0%, *p* = 0.003) and other cancers (44.2% vs. 7.4%, *p* < 0.001). Physically, cases were more likely to engage in low levels of physical activity (82.1% vs. 63.6%, *p* = 0.008). Regarding dietary intake, cases had a significantly higher mean energy intake (2769.64 ± 964.46 vs. 2502.52 ± 932.40 kcal, *p* = 0.04) but a lower dietary fiber intake (27.06 ± 11.71 vs. 48.80 ± 19.85 g, *p* < 0.001). Consequently, cases had significantly lower mean scores for the MD (7.50 ± 2.65 vs. 8.47 ± 2.92, *p* = 0.01), DASH (22.68 ± 4.89 vs. 25.05 ± 4.74, *p* < 0.001), and MIND (6.66 ± 2.17 vs. 7.31 ± 2.09, *p* = 0.03). No significant differences were found for age, weight, BMI, marital status, use of birth control pills, number of children, miscarriage history, or current smoking status.

**Table 1:** Descriptive characteristics of the participants by case–control status.

	Case	Control	*p* value^a^
Age	49.32 ± 9.10	48.84 ± 8.51	0.69
Weight	71.33 ± 15.02	70.67 ± 14.79	0.75
BMI	27.66 ± 5.38	27.49 ± 6.06	0.83
WHR	0.89 ± 0.07	0.83 ± 0.11	**< 0.001**
Education (*n*, %)			**0.04**
Illiterate	10 (9.4%)	5 (4.7%)	
Low	51 (48.1%)	39 (36.4%)	
Medium	22 (20.8%)	22 (20.6%)	
High	23 (21.7%)	41 (38.3%)	
Menopausal (*n*, %)			**< 0.001**
Yes	44 (41.5%)	14 (13.1%)	
No	62 (58.5%)	93 (86.9%)	
Marital status (*n*, %)			0.32
Single	17 (16.2%)	23 (21.5%)	
Married	88 (83.8%)	84 (78.5%)	
Employment status (*n*, %)			**0.002**
Housekeeper	93 (87.7%)	75 (70.1%)	
Employee	13 (12.3%)	32 (29.9%)	
Birth control pills (*n*, %)			0.07
Yes	40 (37.7%)	53 (50.0%)	
No	66 (62.3%)	53 (50.0%)	
Number of children (*n*, %)			0.24
< 5	87 (82.1%)	94 (87.9%)	
≥ 5	19 (17.9%)	13 (12.1%)	
Miscarriage history (*n*, %)			0.05
Yes	24 (24.7%)	15 (14.0%)	
No	73 (75.3%)	92 (86.0%)	
Family history of breast cancer (*n*, %)			**0.003**
Yes	27 (26.0%)	10 (10.0%)	
No	77 (74.0%)	90 (90%)	
Family history of another cancer (*n*, %)			**< 0.001**
Yes	46 (44.2%)	7 (7.4%)	
No	58 (55.8%)	87 (92.6%)	
Current smoker (*n*, %)			0.10
Yes	8 (7.6%)	3 (2.8%)	
No	97 (92.4%)	104 (97.2%)	
Physical activity (MET)^b^ (*n*, %)			**0.008**
Low	87 (82.1%)	68 (63.6%)	
Moderate	17 (16.0%)	32 (29.9%)	
High	2 (1.9%)	7 (6.5%)	
Energy intake	2769.64 ± 964.46	2502.52 ± 932.40	**0.04**
Protein intake	97.97 ± 32.69	89.90 ± 38.97	0.10
Carbohydrate intake	445.48 ± 197.06	397.79 ± 157.56	0.05
Fat intake	72.08 ± 27.48	67.06 ± 30.71	0.21
Dietary fiber intake	27.06 ± 11.71	48.80 ± 19.85	**< 0.001**
MD score	7.50 ± 2.65	8.47 ± 2.92	**0.01**
DASH score	22.68 ± 4.89	25.05 ± 4.74	**< 0.001**
MIND score	6.66 ± 2.17	7.31 ± 2.09	**0.03**

*Note:* Data are presented as mean ± SD or percent (*n*). Significant *p* *‐*values (< 0.05) are highlighted in bold.

Abbreviations: BMI, body mass index; DASH, Dietary Approaches to Stop Hypertension; MD, Mediterranean diet; MIND, Mediterranean‐DASH Intervention for Neurodegenerative Delay diet; WHR, waist‐hip ratio.

^a^Obtained from the independent‐sample *t*‐test, chi‐squared, or Fisher test, where appropriate.

^b^Physical activity based on MET (metabolic equivalent minutes/week).

The characteristics of participants in the lowest (T1) and highest (T3) tertiles of the MD, DASH, and MIND diet scores are shown in Table 2. Participants in the highest tertile of both MD and DASH scores had significantly lower BMI (MD‐T3: 25.75 ± 4.79 vs. MD‐T1: 28.97 ± 6.10, *p* < 0.05; DASH‐T3: 25.70 ± 5.70 vs. DASH‐T1: 28.51 ± 6.33, *p* < 0.05) and WHR (MD‐T3: 0.84 ± 0.09 vs. MD‐T1: 0.87 ± 0.12, *p* < 0.05; DASH‐T3: 0.83 ± 0.08 vs. DASH‐T1: 0.87 ± 0.13, *p* < 0.05) compared to those in the lowest tertile. A consistent and significant finding across all three dietary patterns was that participants in the highest tertile had a substantially higher mean dietary fiber intake compared to those in the lowest tertile (MD: 41.98 ± 19.71 vs. 31.87 ± 17.37, *p* < 0.05; DASH: 44.50 ± 18.37 vs. 32.76 ± 20.61, *p* < 0.05; MIND: 44.69 ± 15.98 vs. 34.25 ± 22.63, *p* < 0.05). For the DASH score, the highest tertile was also associated with significantly lower carbohydrate intake (403.37 ± 187.38 vs. 468.68 ± 177.28, *p* < 0.05). No significant differences were observed across tertiles for age, weight, education, marital status, employment, smoking history, physical activity level, or intakes of energy, protein, and fat.

**Table 2:** Sample characteristics for the lowest (T1) and highest (T3) tertiles of MD, DASH, and MIND scores (*N* = 213)^1^.

	MD score		DASH score		MIND score	
	T1	T3	T1	T3	T1	T3
Age	48.39 ± 9.33	49.54 ± 8.43	48.17 ± 8.70	49.49 ± 8.32	49.39 ± 8.57	49.18 ± 7.42
Weight	72.01 ± 14.31	69.15 ± 14.94	72.51 ± 13.79	70.35 ± 12.79	71.36 ± 13.48	73.34 ± 17.29
BMI	28.97 ± 6.10	25.75 ± 4.79^∗^	28.51 ± 6.33	25.70 ± 5.70^∗^	28.31 ± 6.08	26.77 ± 5.51
WHR	0.87 ± 0.12	0.84 ± 0.09^∗^	0.87 ± 0.13	0.83 ± 0.08^∗^	0.85 ± 0.09	0.85 ± 0.13
Education (*n*, %)						
Illiterate	3 (3.7%)	9 (11.7%)	4 (6.2%)	7 (9.7%)	3 (3.8%)	6 (8.5%)
Low	30 (37.0%)	34 (44.2%)	28 (43.1%)	28 (38.9%)	35 (44.9%)	28 (39.4%)
Medium	19 (23.5%)	12 (15.6%)	11 (16.9%)	15 (20.8%)	15 (19.2%)	18 (25.4%)
High	29 (35.8%)	22 (28.6%)	22 (33.8%)	22 (30.6%)	25 (32.1%)	19 (26.8%)
Marital status (*n*, %)						
Single	14 (17.3%)	16 (21.1%)	15 (23.1%)	11 (15.5%)	19 (24.4%)	11 (15.7%)
Married	67 (82.7%)	60 (78.9%)	50 (76.9%)	60 (84.5%)	59 (75.6%)	59 (84.3%)
Employment status (*n*, %)						
Housekeeper	66 (81.5%)	59 (76.6%)	51 (78.5%)	58 (80.6%)	61 (78.2%)	55 (77.5%)
Employee	15 (18.5%)	18 (23.4%)	14 (21.5%)	14 (19.4%)	17 (21.8%)	16 (22.5%)
Smoking history						
Yes	2 (2.5%)	4 (5.3%)	3 (4.6%)	6 (8.5%)	3 (3.8%)	6 (8.6%)
No	79 (97.5%)	72 (94.7%)	62 (95.4%)	65 (91.5%)	73 (96.2%)	64 (91.4%)
Physical activity (MET)^#^						
Low	61 (75.3%)	155 (72.8%)	55 (84.6%)	155 (72.8%)	64 (82.1%)	155 (72.8%)
Moderate	16 (19.8%)	49 (23.0%)	8 (12.3%)	49 (23%)	11 (14.1%)	49 (23.0%)
High	4 (4.9%)	9 (4.2%)	2 (3.1%)	9 (4.2%)	3 (3.8%)	9 (4.2%)
Energy intake	2651.71 ± 985.71	2587.37 ± 974.24	2846.05 ± 976.19	2565.22 ± 990.37	2803.17 ± 900.20	2550.34 ± 899.79
Carbohydrate	430.15 ± 191.19	411.01 ± 180.19	468.68 ± 177.28	403.37 ± 187.38^∗^	459.60 ± 198.09	402.59 ± 176.42
Protein	93.59 ± 36.62	94.54 ± 39.16	99.91 ± 34.88	89.36 ± 36.31	100.87 ± 36.200	91.03 ± 38.25
Fat	72.27 ± 28.74	76.01 ± 30.42	70.54 ± 28.94	68.28 ± 27.99	66.22 ± 26.53	69.07 ± 28.26
Dietary fiber	31.87 ± 17.37	41.98 ± 19.71^∗^	32.76 ± 20.61	44.50 ± 18.37^∗^	34.25 ± 22.63	44.69 ± 15.98^∗^

Abbreviations: DASH, Dietary Approaches to Stop Hypertension; MD, Mediterranean diet; MIND, Mediterranean‐DASH Intervention for Neurodegenerative Delay diet.

^∗^Significant difference (*p* < 0.05) between the highest (T3) and lowest (T1) tertiles, assessed by an independent‐sample *t*‐test.

The associations between adherence to the MD, DASH, and MIND diets and the odds of breast cancer are presented in Table 3. Among all women, higher adherence to these MD, DASH, and MIND diets was significantly associated with reduced odds of breast cancer in the crude models. In the fully adjusted model, compared to women in the lowest tertile, those in the highest tertile of the MD score had a 75% lower risk of breast cancer (OR = 0.25, 95% CI: 0.10–0.64; *p* trend = 0.005). Similarly, for the DASH score, women in the highest tertile had a 76% lower risk (OR = 0.24, 95% CI: 0.09–0.62; *p* trend = 0.002). The strongest inverse association was observed for the MIND diet, where the highest adherence was associated with a 72% reduction in odds (OR = 0.28, 95% CI: 0.11–0.75; *p* trend = 0.006). When stratified by menopausal status, no statistically significant trends were observed in the premenopausal or postmenopausal subgroups after adjustment for confounders.

**Table 3:** Multivariable‐adjusted odds ratios and 95% confidence intervals for breast cancer across tertiles of MD, DASH, and MIND diet scores.

	Tertiles of dietary score			
Dietary scores	T1	T2	T3	*p* trend
**MD**				
All women				
Crude	1	1.41 (0.73–2.74)	0.45 (0.22–0.90)	**0.03**
Adjusted	1	1.07 (0.45–2.57)	0.25 (0.10–0.64)	**0.005**
Premenopausal				
Crude	1	1.38 (0.63–3.02)	0.46 (0.20–1.07)	0.07
Adjusted	1	1.54 (0.52–4.57)	0.35 (0.11–1.17)	0.59
Postmenopausal				
Crude	1	1.97 (0.41–3.59)	0.56 (0.12–2.48)	0.51
Adjusted	1	0.34 (0.01–7.39)	0.17 (0.06–5.21)	0.27
**DASH**				
All women				
Crude	1	0.63 (0.32–1.22)	0.30 (0.15–0.60)	**0.001**
Adjusted	1	0.58 (0.24–1.39)	0.24 (0.09–0.62)	**0.002**
Premenopausal				
Crude	1	1.19 (0.52–2.69)	0.63 (0.26–1.36)	0.18
Adjusted	1	1.19 (0.41–3.45)	0.42 (0.13–1.35)	0.13
Postmenopausal				
Crude	1	0.25 (0.06–1.07)	0.18 (0.03–1.14)	0.22
Adjusted	1	0.65 (0.42–1.02)	0.58 (0.32–1.07)	0.45
**MIND**				
All women				
Crude	1	0.90 (0.46–1.77)	0.49 (0.25–0.97)	**0.03**
Adjusted	1	1.15 (0.47–2.82)	0.28 (0.11–0.75)	**0.006**
Premenopausal				
Crude	1	1.29 (0.54–3.07)	0.85 (0.37–1.92)	0.58
Adjusted	1	1.73 (0.53–5.61)	0.60 (0.17–2.05)	0.20
Postmenopausal				
Crude	1	0.69 (0.19–2.55)	1.30 (0.12–1.15)	0.91
Adjusted	1	0.93 (0.71–1.21)	0.75 (0.50–1.13)	0.69

*Note:* The logistic regression model, with the lowest tertile as a reference. Adjusted model: adjusted for age, body mass index, waist–hip ratio; physical activity, education level, employment, and family history of breast cancer or other cancer. Significant *p*‐values (< 0.05) are highlighted in bold.

Abbreviations: DASH, Dietary Approaches to Stop Hypertension; MD, Mediterranean diet; MIND, Mediterranean‐DASH Intervention for Neurodegenerative Delay diet.

## 9. Discussion

The present study was aimed at investigating the association between adherence to the MD, DASH, and MIND diet scores and the odds of breast cancer among Iranian women. Our findings revealed that greater adherence to these dietary patterns was associated with significantly lower odds of breast cancer. Specifically, the highest adherence to the MD, DASH, and MIND diets was linked to a 75%, 76%, and 72% reduction in odds of breast cancer, respectively. These results underscore the potential role of diet in mitigating breast cancer risk and offer insights into the relevance of these dietary patterns in the context of Iranian women. This population has been underrepresented in previous studies.

When comparing our findings with the existing literature, we found strong support from similar studies. For example, a previous case–control study among Iranian women by Aghamohammadi et al. similarly reported an inverse association between MIND diet adherence and breast cancer risk [10]. Our findings directly confirm this association in the Iranian population, reinforcing the potential protective role of the MIND diet in this specific demographic. Furthermore, by simultaneously investigating and identifying similar inverse associations for the MD and DASH diets, our study extends the evidence base, suggesting that multiple healthy dietary patterns, which share core components such as high intakes of fruits, vegetables, and whole grains, may be beneficial for breast cancer prevention in Iranian women. Similarly, a meta‐analysis confirmed the inverse relationship between adherence to the MD and breast cancer risk, reinforcing our findings related to the MD [6]. These studies are consistent with our results, suggesting that the dietary patterns examined in our study may be protective against breast cancer across different populations. However, contrasting findings have also been reported in the literature. For instance, a case–control study examining the association between MIND diet adherence and the odds of breast cancer did not find a significant protective effect [11], which contrasts with our findings. One possible explanation for this discrepancy could be the differences in study design and sample characteristics. The case–control study included a larger sample and spanned a broader age range, which might have influenced the strength of the observed association due to age‐related variations in dietary effects.

Our findings suggest that adherence to healthy dietary patterns such as MD, DASH, and MIND may play a role in reducing the risk of breast cancer. The mechanisms underlying the observed association between adherence to the MD, DASH, and MIND diets and reduced odds of breast cancer risk are complex and multifactorial. Several potential biological pathways may explain these findings. These dietary patterns are rich in anti‐inflammatory foods, including fruits, vegetables, whole grains, and healthy fats (such as olive oil in the MD), which can help reduce systemic inflammation. Chronic inflammation is recognized as a key factor in the development and progression of various cancers, including breast cancer. Therefore, the anti‐inflammatory properties of these diets could contribute to their protective effects [17].

Additionally, these diets are high in antioxidants, particularly vitamins C and E, polyphenols, and flavonoids, which are abundant in fruits, vegetables, and nuts. These antioxidants help neutralize reactive oxygen species, thereby preventing oxidative DNA damage, which can lead to mutations and carcinogenesis [17]. The consumption of fiber, which is prominent in all three dietary patterns, also plays a significant role. In our study, controls had a substantially higher mean fiber intake than cases (48.80 vs. 27.06 g/day; *p* < 0.001). Fiber has been shown to modulate estrogen metabolism by promoting the excretion of estrogen metabolites and favorably altering the gut microbiota, thus reducing the exposure of breast tissue to estrogens, which is a well‐established risk factor for breast cancer [18]. Furthermore, the MD and DASH diets, by focusing on healthy fats and lean proteins, promote a favourable lipid profile that may reduce the production of tumor‐promoting hormones, such as insulin and insulin‐like growth factors [17, 19].

Another possible mechanism specific to the MIND diet, which emphasizes brain‐healthy foods, is its potential influence on the gut–brain axis and its role in modulating the immune system. The consumption of foods rich in omega‐3 fatty acids (e.g., fish), which is emphasised in the MIND diet, may have a protective effect by enhancing immune surveillance and promoting an anticarcinogenic microenvironment [20]. Additionally, the MIND diet′s emphasis on berries and green, leafy vegetables, both of which are high in bioactive compounds, may also contribute to cancer prevention by modulating cellular signalling pathways involved in cell proliferation and apoptosis [19].

The practical implications of this research are significant, as it provides further evidence to support the promotion of healthy dietary patterns as part of cancer prevention strategies, particularly in populations at high risk for breast cancer. Public health campaigns that encourage adherence to the MD, DASH, and MIND diets could potentially reduce the incidence of breast cancer. Moreover, these findings may inform dietary guidelines in Iran and other Middle Eastern countries, where nutritional habits differ significantly from those of Western populations. Future research should focus on conducting prospective cohort studies to establish causal relationships between dietary adherence and breast cancer risk. Additionally, studies exploring the biological mechanisms underlying these associations, such as the impact of diet on gene expression and immune function, would provide valuable insights into how these diets exert their protective effects. Investigating the role of specific food components within these dietary patterns, such as antioxidants and fiber, could also help refine nutritional recommendations for cancer prevention.

Our findings provide support for the role of these dietary patterns in reducing the odds of breast cancer, yet it is essential to consider the broader context. The ability of these diets to minimise cancer risk likely depends not only on the specific foods consumed but also on their overall nutrient composition and their effects on metabolic and immune pathways that influence cancer progression [19]. Furthermore, lifestyle factors such as physical activity, weight management, and socioeconomic status, which were also assessed in this study, can interact with diet to influence breast cancer risk [21]. Future studies should explore the combined effects of diet and lifestyle interventions on breast cancer prevention.

Nevertheless, the study has several limitations that must be considered. First, the case–control design of our study limits the ability to infer causality between diet and breast cancer risk. While our results suggest an association, prospective studies are needed to confirm whether adherence to these diets directly influences breast cancer development. Second, the study relied on self‐reported dietary intake, which is inherently subject to recall bias and measurement error. Although the FFQ used in this study has been validated in the Iranian population, dietary habit misreporting remains a possibility. Third, the sample was drawn from a single tertiary hospital in Ahvaz, an urban center in southwest Iran. This may limit the generalizability of our findings to women from rural areas or other Iranian regions with different socioeconomic, cultural, and dietary backgrounds. Future studies with larger, more diverse samples are necessary to validate these findings and explore the generalizability of the results.

In conclusion, our study provides compelling evidence that adherence to the MD, DASH, and MIND diets is associated with reduced odds of breast cancer in Iranian women. These findings contribute to the growing body of literature on the role of diet in cancer prevention and underscore the importance of promoting healthy dietary patterns as part of public health strategies to reduce breast cancer risk.

## Author Contributions

The authors′ responsibilities were as follows: F.B. and H.R.: conceived and designed the study; N.M. and A.H. collected the data; F.B. and M.R.Z.: analyzed data or performed statistical analyses; F.B. and M.M.: wrote the manuscript; A.Z.J. and S.R.: critically revised the manuscript for important intellectual content; and H.R.: had primary responsibility.

## Funding

This work was supported by the Ahvaz Jundishapur University of Medical Sciences, 10.13039/501100005001, 02s53.

## Disclosure

All authors read and approved the final manuscript.

## Ethics Statement

This study was conducted in accordance with the guidelines outlined in the Declaration of Helsinki, and all procedures involving human subjects were approved by the Ethics Committee of the Ministry of Health and Medical Education of Iran and the Ahvaz Jundishapur University of Medical Sciences (Approval Number IR.AJUMS.REC.1402.386). Written informed consent was obtained from all subjects.

## Consent

The authors have nothing to report.

## Conflicts of Interest

The authors declare no conflicts of interest.

## Data Availability

The data that support the findings of this study are available from the corresponding author upon reasonable request.
